# Functional Analysis of Rift Valley Fever Virus NSs Encoding a Partial Truncation

**DOI:** 10.1371/journal.pone.0045730

**Published:** 2012-09-19

**Authors:** Jennifer A. Head, Birte Kalveram, Tetsuro Ikegami

**Affiliations:** 1 Department of Microbiology and Immunology, The University of Texas Medical Branch, Galveston, Texas, United States of America; 2 Department of Pathology, The University of Texas Medical Branch, Galveston, Texas, United States of America; 3 Sealy Center for Vaccine Development, The University of Texas Medical Branch, Galveston, Texas, United States of America; 4 Center for Biodefense and Emerging Infectious Diseases, The University of Texas Medical Branch, Galveston, Texas, United States of America; George Mason University, United States of America

## Abstract

Rift Valley fever virus (RVFV), belongs to genus *Phlebovirus* of the family *Bunyaviridae*, causes high rates of abortion and fetal malformation in infected ruminants as well as causing neurological disorders, blindness, or lethal hemorrhagic fever in humans. RVFV is classified as a category A priority pathogen and a select agent in the U.S., and currently there are no therapeutics available for RVF patients. NSs protein, a major virulence factor of RVFV, inhibits host transcription including interferon (IFN)-β mRNA synthesis and promotes degradation of dsRNA-dependent protein kinase (PKR). NSs self-associates at the C-terminus 17 aa., while NSs at aa.210–230 binds to Sin3A-associated protein (SAP30) to inhibit the activation of IFN-β promoter. Thus, we hypothesize that NSs function(s) can be abolished by truncation of specific domains, and co-expression of nonfunctional NSs with intact NSs will result in the attenuation of NSs function by dominant-negative effect. Unexpectedly, we found that RVFV NSs truncated at aa. 6–30, 31–55, 56–80, 81–105, 106–130, 131–155, 156–180, 181–205, 206–230, 231–248 or 249–265 lack functions of IFN–β mRNA synthesis inhibition and degradation of PKR. Truncated NSs were less stable in infected cells, while nuclear localization was inhibited in NSs lacking either of aa.81–105, 106–130, 131–155, 156–180, 181–205, 206–230 or 231–248. Furthermore, none of truncated NSs had exhibited significant dominant-negative functions for NSs-mediated IFN-β suppression or PKR degradation upon co-expression in cells infected with RVFV. We also found that any of truncated NSs except for intact NSs does not interact with RVFV NSs even in the presence of intact C-terminus self-association domain. Our results suggest that conformational integrity of NSs is important for the stability, cellular localization and biological functions of RVFV NSs, and the co-expression of truncated NSs does not exhibit dominant-negative phenotype.

## Introduction

Rift Valley fever virus (RVFV) belongs to genus *Phlebovirus* of the family *Bunyaviridae*, and is a mosquito-borne zoonotic pathogen which causes Rift Valley fever (RVF). RVF is characterized by an acute febrile illness, hemorrhagic fever, neurological disorder or blindness in humans [1], [2], [3], [4]. In ruminants, RVFV induces a high rate of abortion or fetal malformation as well as lethal hepatitis in newborn lambs [5]. The first recognized outbreak of RVF occurred in Kenya in 1930 [6], and RVFV has spread from endemic region in sub-Saharan Africa into Egypt [7], Madagascar and the Arabian Peninsula [8], [9], [10], [11], [12]. The potential threat of RVFV introduction into non-endemic countries raises concern of agriculture and public health [13], [14], [15]. RVFV is a risk group 3 pathogen, Category A pathogen and an overlap select agent by the CDC/USDA [16]. The handling of wild-type (wt) RVFV within the U.S. requires BSL3+ or BSL4 facilities. Live-attenuated MP-12 vaccine strain is excluded from select agent rule, and handled at BSL2 level. MP-12 encodes for functional NSs protein, which is useful for the analyses of various NSs functions at BSL2 level [17], [18], [19].

RVFV has a tripartite negative-stranded RNA genome, referred to as Small (S)-, Medium (M)- and Large (L)-segment. The S-segment encodes for N and NSs genes in an ambi-sense manner, M-segments encodes for NSm, 78-kD protein, NSm-Gn, Gn, and Gc proteins, and L-segment encodes for RNA-dependent RNA polymerase [20], [21], [22], [23]. NSs is a major virulence factor of RVFV and inhibits host general transcription through sequestration of TFIIH p44 [24] or promotion of TFIIH p62 subunits degradation [19]. NSs also inhibits host antiviral response by inhibiting the activation of interferon (IFN)-β promoter through interaction with Sin3A-associated protein (SAP30) at aa.210–230 [25], [26], and promotion of dsRNA-dependent protein kinase (PKR) degradation [17], [27], [28].

Developing countermeasures against RVFV is important for the prevention of RVF outbreaks or decreasing impact of RVFV introduction. A number of candidate vaccines are under development including live-attenuated vaccine [29], [30], [31], [32], [33], [34], [35], [36], [37], [38], [39], [40], formalin-inactivated vaccine [41], [42], subunit vaccine [43], virus-like particle [44], [45], [46], [47], nonspreading RVFV replicon [48], [49], viral vectors [50], [51], [52], [53], [54], [55], [56], and DNA vaccines [57], [58], [59]. For treatment of ongoing outbreaks, several antivirals have been tested for RVFV infection. Liposome-encapsulated ribavirin is effective to treat RVFV infection in mice [60], while a potent IFN inducer, polyriboinosinic-polyribocytidylic acid stabilized with poly-L-Lysine and carboxymethyl cellulose [Poly(LCIC)] are effective in combination with ribavirin [61]. Therapeutic administration of IFN-α into rhesus monkeys infected with RVFV also limits RVFV replication [62]. The 6-fluoro-3-hydroxy-2-pyrazinecarboxamide (T-705) is shown to be more effective to inhibit RVFV replication than ribavirin [63], while antiviral small molecule, LJ001 was shown to be effective to numerous enveloped viruses including RVFV [64]. These studies suggest that increased innate immune responses could inhibit RVFV replication in addition to antivirals specific to viral proteins.

Since the major virulence factor, NSs protein, is an antagonist of IFN responses, the direct attenuation of NSs function may increase host innate immune responses in cells infected with RVFV potentially limiting RVFV replication. Thus, we aimed to attenuate RVFV NSs function(s) by co-expressing nonfunctional NSs. It has been shown that recombinant MP-12 virus encoding truncated NSs at aa.210 to 230 (SAP30-binding domain) does not inhibit IFN-β mRNA synthesis [26]. Thus, we employed a similar strategy to abolish a part of NSs functions by truncating each 17 to 25 aa. Co-expression of such truncated NSs may exhibit dominant-negative phenotype by self-association through the C-terminus at the self-association domain (aa.249 to 265) [65]. In this study, we generated NSs encoding deletions of 17 to 25 amino acids, characterized the functions of truncated NSs, and analyzed the dominant-negative effects of co-expressed truncated NSs in cells infected with RVFV.

## Results

### Generation of recombinant MP-12 encoding NSs encoding a 17 to 25 aa. truncation

**Figure 1:**
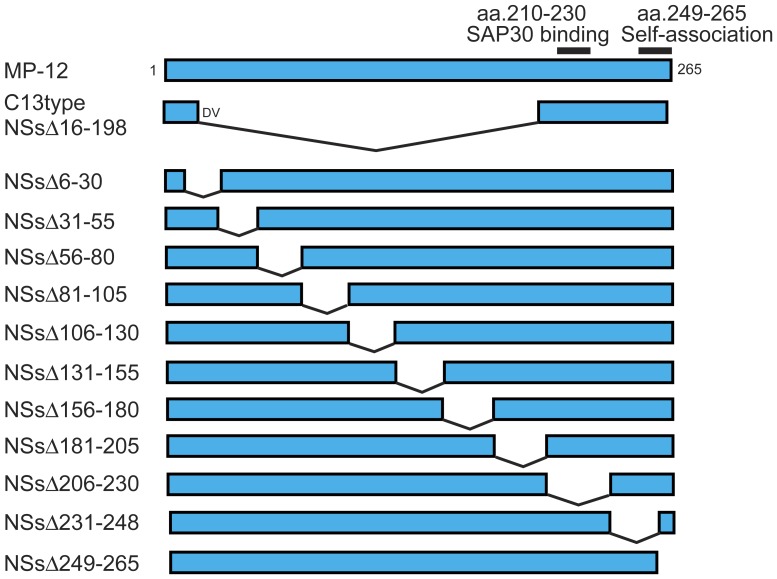
Schematics of truncated NSs design. MP-12 NSs (top) encodes SAP30-binding domain (aa.210–230)[26] and C-terminus self-association domain (aa.249–265)[65]. The rMP12-C13type (C13type) encodes an in-frame truncation of aa.16–198, and the N-terminus and C-terminus fragments are linked with Asp (D) and Val (V). Other 11 NSs mutants encode in-frame 25 or 17 amino acid truncations.

**Figure 2:**
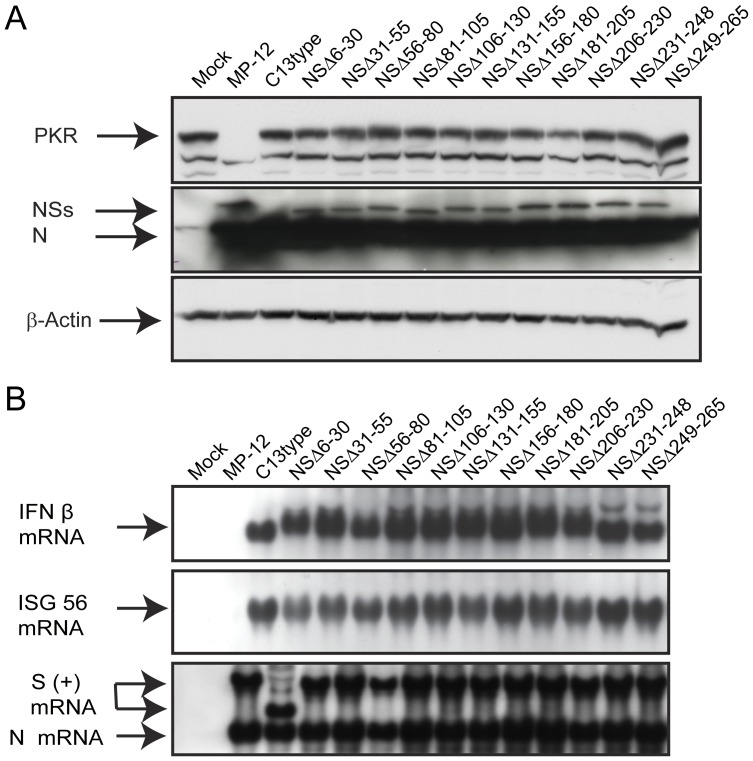
Functions of truncated NSs (A) VeroE6 cells were mock-infected or infected with indicated viruses at an moi of 3. Cells were collected at 16 hpi, and the abundance of PKR (anti-PKR antibody), NSs and N (anti-RVFV antibody) and β-actin (anti-actin antibody) were analyzed by Western blot. (B) A549 cells were mock-infected or infected with indicated viruses at an moi of 3. Total RNA was extracted at 7 hpi, and IFN-β mRNA, ISG56 mRNA and RVFV anti-viral-sense S-RNA/N mRNA were detected by Northern blot with specific RNA probe [66], [67]. Representative data from at least 3 independent experiments are shown.

Using reverse genetics for the RVFV MP-12 strain, we recovered 11 recombinant MP-12 viruses which encode NSs protein with a 17 to 25 aa. truncation (**Fig. 1**). These NSs mutants exhibited different plaque phenotypes in plaque assay (**Fig. S1**) suggesting possible variation of attenuation by each NSs mutant. The plaques of NSΔ6–30 and NSΔ56–80 were clear in neutral red stain, while other mutants made turbid plaques. To test the functions of each NSs protein, VeroE6 cells (type-I IFN-incompetent) were mock-infected or infected with MP-12, rMP12-C13type (a control lacking NSs functions)[18], and NSs mutants using an moi of 3. After 16 hours, cells were collected and the abundance of PKR was measured (**Fig. 2A**) as described previously [17]. As expected, PKR was not detectable in cells infected with MP-12 by post-translational downregulation [17], but was detected in cells mock-infected or infected with rMP12-C13type. However, cells infected with the MP-12 encoding partially truncated NSs did not decrease PKR abundance.

Next, we tested if partial deletions within the NSs gene would affect the inhibition of IFN-β mRNA synthesis. Type-I IFN-competent A549 cells were mock-infected or infected with MP-12, rMP12-C13type or NSs mutants at an moi of 3, and then total RNA was extracted at 7 hpi. Northern blot was performed using RNA probe specific to human IFN-β, ISG56 or RVFV anti-viral-sense S RNA/N mRNA as described previously [66], [67]. We tested ISG56 gene, one of the genes controlled under IFN-stimulated response element (ISRE), to confirm the inhibition of host transcription suppression including IFN-β mRNA by NSs. As expected, cells infected with MP-12 inhibited the synthesis of IFN-β and ISG56 mRNA, while those infected with rMP12-C13type induced both IFN-β and ISG56 mRNA (**Fig. 2B**). Interestingly, none of NSs mutants, including NSΔ210–230 lacking SAP30-binding domain, had inhibited IFN-β mRNA synthesis. Viral replication of those mutants was significantly decreased in type-I IFN-competent MRC-5 cells (**Fig. S2**). Therefore, it was concluded that a series of MP-12 encoding partially truncated NSs gene does not degrade PKR and inhibit IFN-β mRNA synthesis.

**Figure 3:**
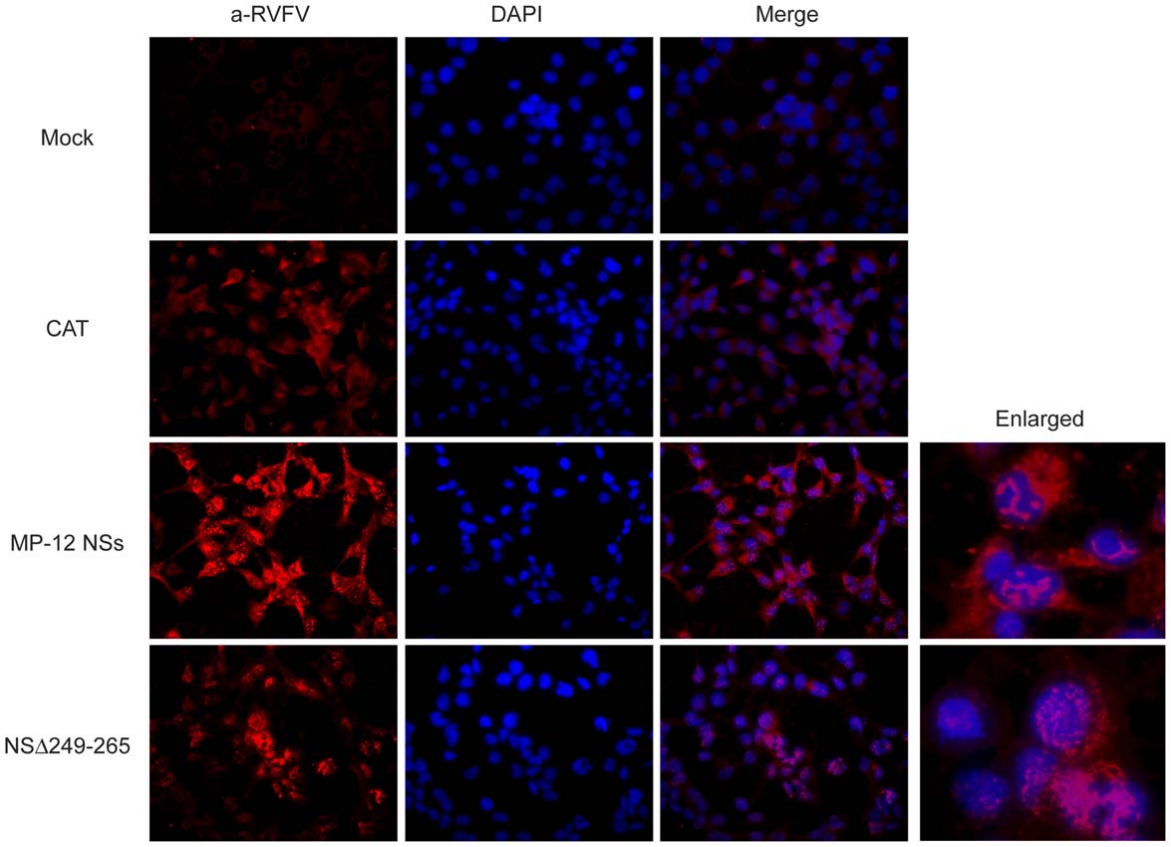
NSs expression of MP-12 and NSΔ249 –**265.** 293 cells were mock-transfected (mock) or transfected with in vitro transcribed RNA encoding CAT (control) or NSs of MP-12 or NSΔ249–265. At 16 hpi, cells were fixed with methanol for 5 min, and stained with anti-RVFV antibody (1:500) at 37^°^C for 1 hr and Alexa Fluor 594, goat anti-mouse IgG (H+L) at 37^°^C for 1 hr. After washing with PBS, cells were stained with DAPI, and observed under fluorescent microscope.

In Western blot as shown in **Fig. 2A**, all NSs mutants, except for NSΔ249–265, could be detectable by using anti-RVFV mouse polyclonal antibody. It was possible the anti-RVFV polyclonal antibody does not sufficiently contain antibodies reactive to linear epitopes except for the C-terminus. Thus, we next tested the accumulation of NSΔ249–265 by using indirect immunofluorescent assay to know if the same anti-RVFV polyclonal antibody can recognize conformational epitopes on NSΔ249–265. 293 cells were transfected with *in vitro* synthesized RNA encoding NSs of MP-12, NSΔ249–265 or chloramphenicol acetyltransferase (CAT) (control), and the cells were fixed with methanol at 16 hours post transfection. Nuclear filamentous inclusion was observed in cells expressing NSs of MP-12 or NSΔ249–265, while the specific signals of NSs accumulation were weaker in cells expressing NSΔ249–265 than those expressing MP-12 NSs (**Fig. 3**). NSΔ249–265 was also accumulated in cytoplasm.

**Figure 4:**
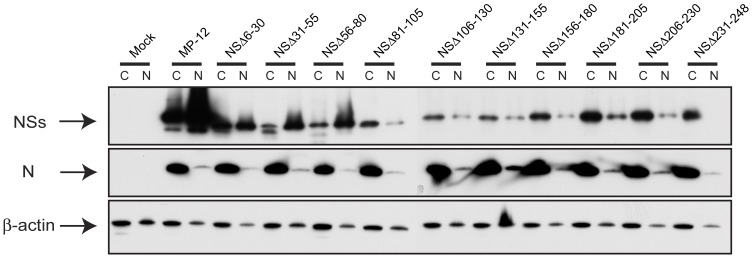
Cellular localization of truncated NSs. 293 cells were mock-infected or infected with MP-12 or recombinant MP-12 encoding partially truncated NSs at an moi of 3. Cell lysates were collected at 16 hpi, and nuclear and cytoplasmic fractions were separated as described in materials and methods. RVFV NSs, N and β-actin were detected by Western blot.

Next, we tested the cellular localization of truncated NSs other than NSΔ249–265 by Western blot (**Fig. 4**). We did not include NSΔ249–265 for the experiment as no antibodies were available to detect this NSs in Western blot. 293 cells were mock-infected or infected with MP-12 or NSs truncation mutants at an moi of 3. Cells were collected at 16 hpi, and nuclear and cellular fractions were analyzed for the presence of NSs proteins. MP-12 NSs were accumulated both at cytoplasm and nucleus, while N proteins were exclusively localized at cytoplasm, which is consistent with previous study [65]. Abundant accumulation of NSs in nucleus was only observed in cells infected with NSsΔ6–30, NSsΔ 31–55 and NSsΔ56–80, while other mutants, NSsΔ 81–105, NSsΔ106–130, NSsΔ131–155, NSsΔ156–180, NSsΔ181–205 and NSsΔ206–230, NSsΔ231–248 poorly accumulated NSs in nucleus.

### Co-expression of nonfunctional truncated NSs in cells infected with MP-12

To find if co-expression of nonfunctional NSs is able to attenuate PKR degradation function of MP-12 NSs, VeroE6 cells were infected with MP-12 and then co-infected with rMP12-C13type or one of the NSs truncation mutants using an moi of 3. Cells were collected at 16 hpi and Western blot was used to measure abundance of PKR and RVFV NSs. However, it was found that levels of MP-12 NSs accumulation were not identical to those expressing truncated NSs (**Fig. S3**). Only cells co-infected with NSΔ6–30 and NSΔ56–80 allowed an efficient accumulation of MP-12 NSs. As a result, PKR was abundantly detected in cells infected with MP-12 and NSs mutants.

**Figure 5:**
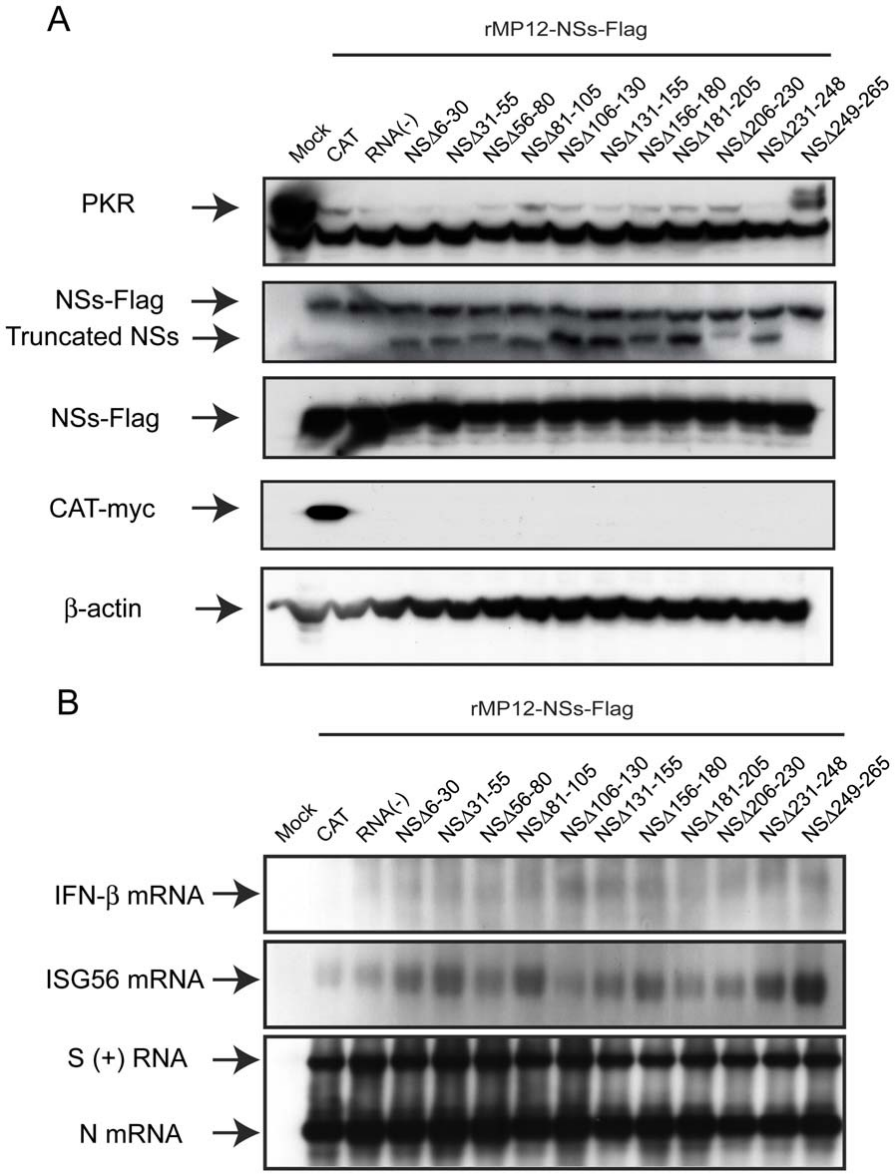
Co-expression of truncated NSs in RVFV-infected cells. 293 cells were mock-infected or infected with rMP12-NSs-Flag at an moi of 3, and mock-transfected or immediately transfected with in vitro transcribed RNA encoding CAT (control) or NSs with indicated truncations. Cells were collected at 16 hpi, and PKR (anti-PKR antibody), NSs (anti-RVFV antibody), NSs-Flag (anti-Flag antibody), CAT-myc (anti-myc antibody) and β-actin (anti-actin antibody) were detected by Western blot. (B) A549 cells were mock-infected or infected with rMP12-NSs-Flag at an moi of 3, and mock-transfected or immediately transfected with in vitro transcribed RNA encoding CAT or NSs with indicated truncations. Total RNA was extracted at 7 hpi, and IFN-β mRNA, ISG56 mRNA and RVFV anti-viral-sense S-RNA/N mRNA were detected by Northern blot with specific RNA probe. Representative data from at least 3 independent experiments are shown.

We attempted to allow accumulation of MP-12 NSs by using 293 cells. Cells were mock-infected or infected with rMP12-NSs-Flag, which encode Flag-tagged NSs in place of intact NSs, at an moi of 3, and subsequently mock-transfected (a control) or immediately transfected with *in vitro* synthesized RNA encoding CAT (a control), or NSs mutants, as described previously [17]. Cells were collected at 16 hpi, and the abundance of PKR and RVFV proteins were analyzed by Western blot. All cells infected with rMP12-NSs-Flag accumulated abundant levels of NSs (**Fig. 5A**). PKR was degraded in infected cells mock-transfected or transfected with CAT RNA, while PKR was also degraded in infected cells transfected with RNA encoding NSΔ6–30, NSΔ31–55, NSΔ56–80, NSΔ81–105, NSΔ106–130, NSΔ131–155, NSΔ156–180, NSΔ181–205, NSΔ206–230 or NSΔ231–248. On the other hand, the expression of NSΔ249–265 lacking the C-terminus self-association domain slightly increased the abundance of PKR in cells infected with rMP12-NSs-Flag.

We next tested the effect of co-expression of NSs mutants in the inhibition of IFN-β mRNA synthesis by MP-12 NSs. A549 cells were mock-infected or infected with rMP12-NSs-Flag using moi of 3 and then either mock-transfected or immediately transfected with *in vitro* synthesized RNA encoding CAT or NSs mutants. Total RNA was extracted at 7 hpi, and Northern blot was performed as described above. None of cells transfected with RNA encoding NSs mutants increased the synthesis of IFN-β mRNA (**Fig. 5B**). On the other hand, cells transfected with RNA encoding NSΔ249–265 slightly increased ISG56 mRNA abundance (**Fig. 5B**). These results suggest that nonfunctional NSs encoding the C-terminus self- association domain do not have dominant-negative function, while those lacking the C-terminus domain slightly inhibit PKR degradation as well as ISG56 mRNA synthesis.

**Figure 6:**
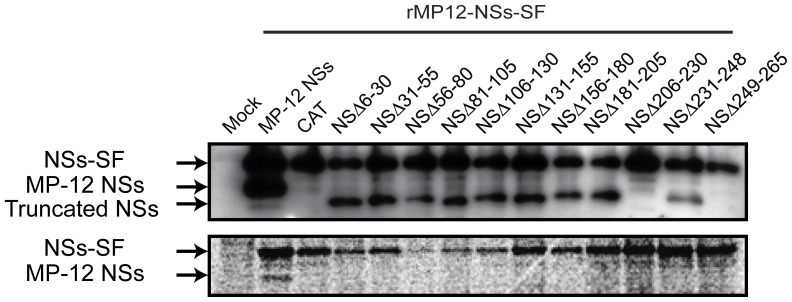
Association of MP-12 NSs and truncated NSs. 293 cells were mock-infected or infected with rMP12-NSs-SF at an moi of 3, and cells were immediately transfected with in vitro synthesized RNA encoding MP-12 NSs, CAT, or truncated NSs. After incubation for 6 hours, newly synthesized proteins were then labeled with [^35^S] methionine/cycteine. Using cell lysates, SF-tagged proteins were precipitated with Strep-Tactin beads. Then, co-precipitated proteins were analyzed by separating on 10% SDS-PAGE gel and followed by autoradiography. Part of lysates before precipitation were used for Western blot using anti-NSs antibody (input control).

Co-affinity precipitation studies were conducted with use of Strep-tagged protein purification to know if over-expressed truncated NSs can interact with MP-12 NSs in infected cells. 293 cells were infected with moi 3 of rMP-12-NSs-SF (recombinant virus tagged with tandem Strep-Tag and Flag)[19] and were then transfected using the *in vitro* synthesized capped RNA encoding each of the truncated NSs mutants. After 6 hours, newly synthesized host and viral proteins were labeled with [^35^S] methionine/cysteine for 4 hours. Whole cell lysates were mixed with Strep-Tactin beads, and SF-tagged MP-12 NSs and bound host and viral proteins were precipitated. Presence of NSs bands were visualized with autoradiography (**Fig. 6**). As expected, MP-12 NSs was co-precipitated with NSs-SF, and MP-12 NSs was migrating slightly faster than that of NSs-SF. The expression of truncated NSs was lower than that of MP-12 NSs (see input), while the expression of NSsΔ206–230 was not detectable. The same phenomenon was observed in a repeated experiment, suggesting the instability of NSsΔ206–230 expression in this experiment. On the other hand, we could not detect co-precipitation of any truncated NSs with NSs-SF. Collectively, our results suggest that those truncated NSs accumulates in cells at low level, mislocalizes, and do not interrupt the MP-12 NSs functions by co-expression.

### Generation of nonfunctional NSs with point mutations at C-terminus

**Figure 7:**
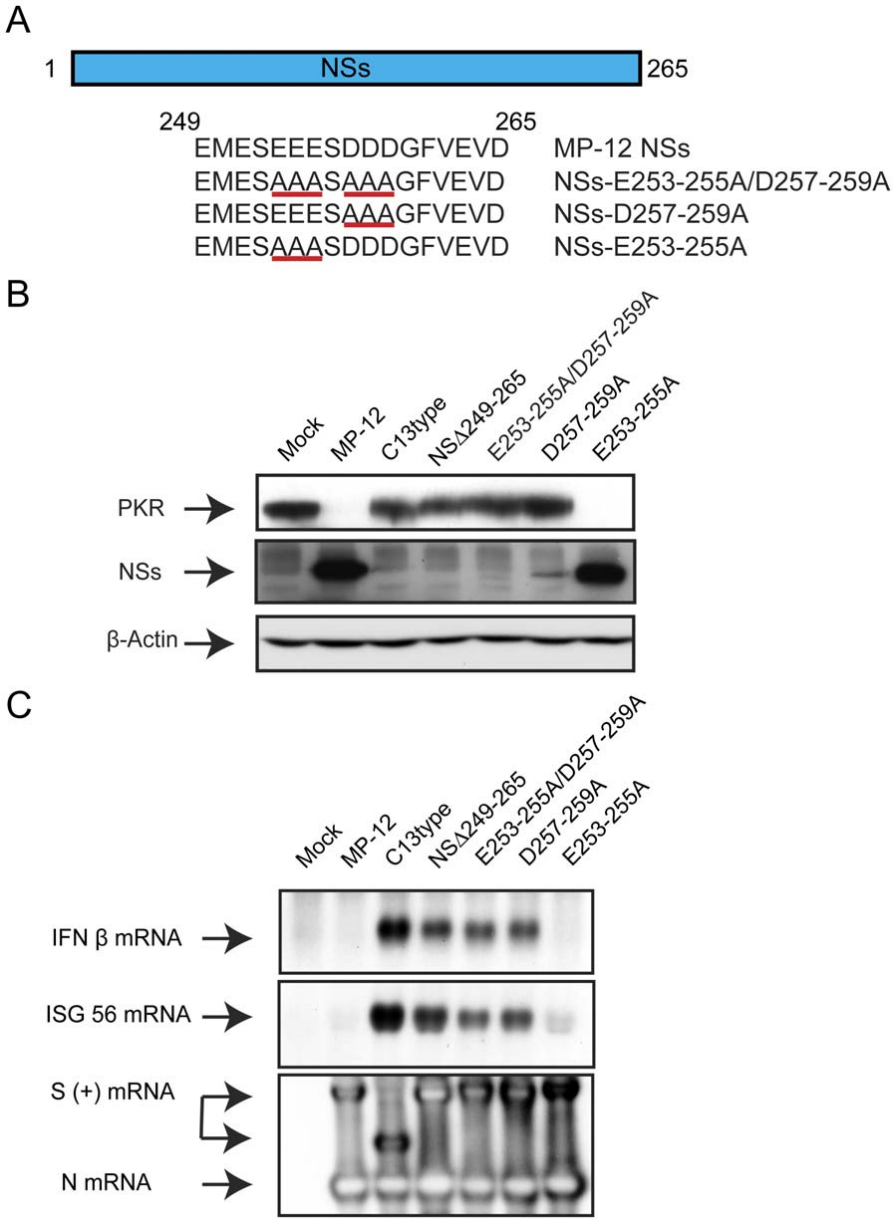
Characterization of NSs encoding alanine substitutions at the C-terminus. (A) Schematics of NSs mutations. Acidic residues, Glu (E) at aa.263–255 and Asp (D) at aa.257–259, were substituted with alanine, and recombinant MP-12 encoding NSs mutations were recovered (NSs-E253-255A/D257–259, NSs-D257-259A and NSs-E253-255A). Underline indicates the amino acids substituted. (B) VeroE6 cells were mock-infected or infected with indicated viruses at an moi of 3. Cells were collected at 16 hpi, and the abundance of PKR (anti-PKR antibody), NSs and N (anti-RVFV antibody), and β-actin (anti-actin antibody) were analyzed by Western blot. (B) A549 cells were mock-infected or infected with indicated viruses at an moi of 3. Total RNA was extracted at 7 hpi, and IFN-β mRNA, ISG56 mRNA and RVFV anti-viral-sense S-RNA/N mRNA were detected by Northern blot with specific RNA probe. Representative data from at least 3 independent experiments are shown.

As NSs lacking C-terminus exhibited slight dominant-negative effect on PKR degradation, we hypothesized that an intact sequence at aa.1 to 248 is required for the dominant-negative effect. We next tested the effect of the C-terminus on the dominant-negative effect. We substituted two sequential acidic amino acids triplets located at the C-terminus with alanines as shown in **Fig. 7A**; i.e., NSs-E253-255A/D257-259A, NSsD257-259A or NSs-E253-255A. We found that NSs-E253-255A could form filamentous inclusion bodies (data not shown), and accumulation was equivalent to that of MP-12 NSs (**Fig. 7B**). On the other hand, NSs-E253-255A/D257-259A or NSsD257-259A did not accumulate in cells efficiently, and the NSs could not be detected with IFA (data not shown). We then tested PKR degradation function and IFN-β mRNA suppression function of those mutants using VeroE6 cells and A549 cells using the same method as described above. Cells infected with NSs-E253-255A degraded PKR (**Fig. 7B**), and inhibited the synthesis of IFN-β mRNA (**Fig. 7C**), while those infected with NSs-E253-255A/D257-259A or NSsD257-259A did not degrade PKR and did not inhibit IFN-β mRNA synthesis. The results suggest that the glutamic acid at aa.253 to 255 can be replaced without affecting NSs functions, while aspartic acid at aa.257 to 259 is important for NSs stability.

### Co-expression of nonfunctional NSs with point mutations at the C-terminus in cells infected with MP-12

**Figure 8:**
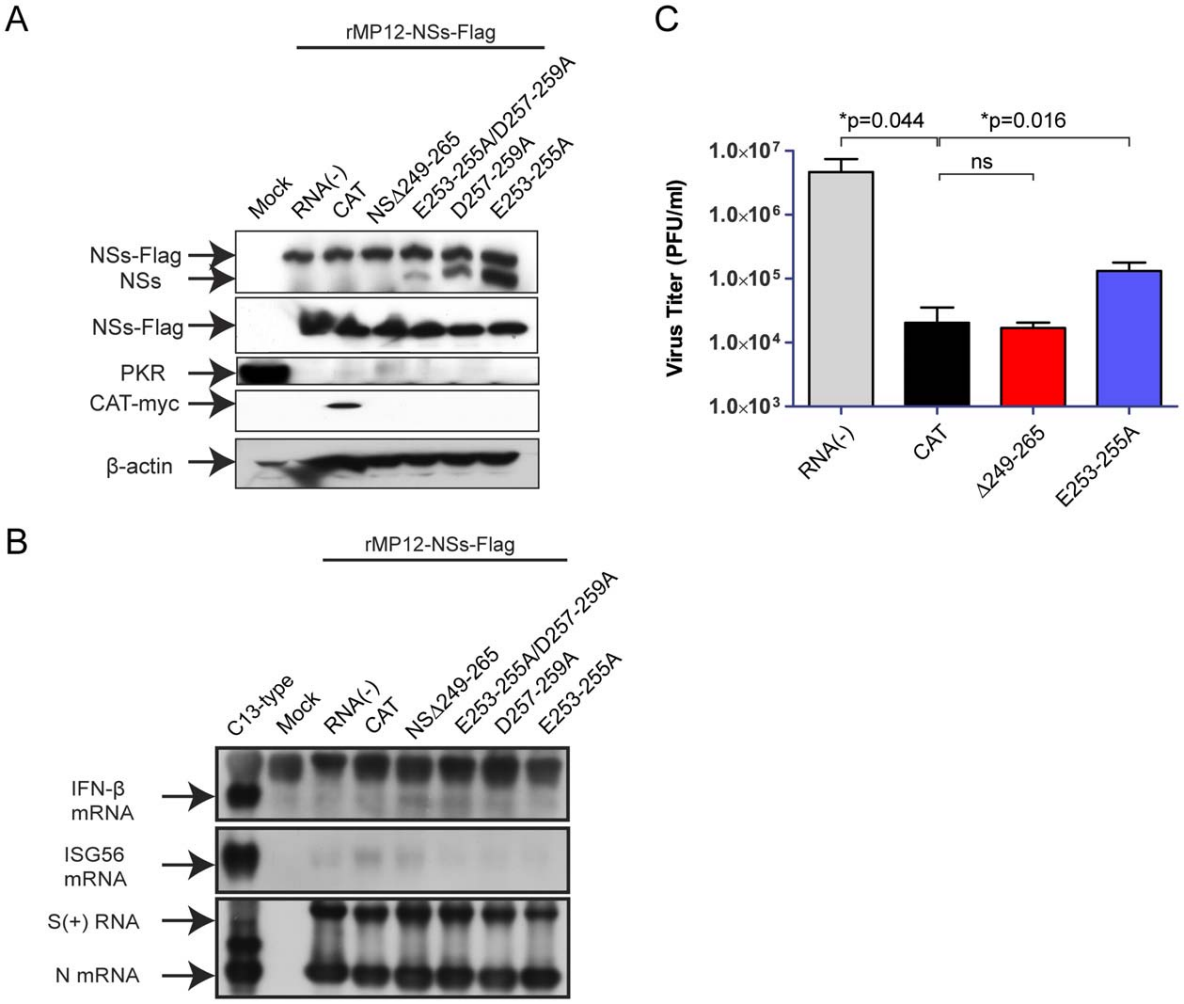
Co-expression of truncated NSs in RVFV-infected cells. 293 cells were mock-infected or infected with rMP12-NSs-Flag at an moi of 3, and mock-transfected or immediately transfected with in vitro transcribed RNA encoding CAT (control) or NSs with indicated NSs mutants. Cells were collected at 16 hpi, and NSs-Flag/NSs (a-RVFV antibody), NSs-Flag (a-Flag antibody), PKR (anti-PKR antibody), CAT-myc (anti-myc antibody) and β-actin (anti-actin antibody) were detected by Western blot. (B) A549 cells were mock-infected or infected with rMP12-NSs-Flag at an moi of 3, and mock-transfected or immediately transfected with in vitro transcribed RNA encoding CAT or NSs with indicated NSs mutants. As a control to induce IFN-β and ISG56 mRNA synthesis, A549 cells were infected with rMP12-C13type (C13type) at an moi of 3. Total RNA was extracted at 7 hpi, and IFN-β mRNA, ISG56 mRNA and RVFV anti-viral-sense S-RNA/N mRNA were detected by Northern blot with specific RNA probe. Representative data from at least 3 independent experiments are shown. (C) A549 cells were infected with MP-12 at moi of 0.01, and mock-transfected or immediately transfected with in vitro transcribed RNA encoding CAT or NSs of NSΔ249–265 or NSs-E253-255A. Culture supernatants were harvested at 72 hpi, and virus titers were measured by plaque assay. P-values of unpaired Student's t-test are shown (*; p<0.05, ns; not significant).

We next tested the effect of co-expression of NSs-E253-255A/D257-259A, NSsD257-259A or NSs-E253-255A in infected cells, as described above. 293 cells were mock-infected or infected with rMP12-NSs-Flag using a moi of 3, then cells were mock-transfected or transfected with *in vitro* synthesized RNA encoding CAT, NSΔ249–265, NSs-E253-255A/D257-259A, NSsD257-259A or NSs-E253-255A. Cells were collected at 16 hpi for Western blot analysis. As shown in **Fig. 8A**, co-expression of NSs-E253-255A/D257-259A or NSs-D257-259A or NSs-E253-255A did not inhibit PKR degradation by rMP12-NSs-Flag, while that of NSΔ249-265 very slightly increased the PKR abundance, which is consistent with the result in **Fig. 5A**.

To test the co-expression effect of those NSs mutants in IFN-β mRNA synthesis, A549 cells were mock-infected or infected with rMP12-NSs-Flag at a moi of 3, and immediately transfected with *in vitro* synthesized RNA encoding NSs-E253-255A/D257-259A, NSsD257-259A or NSs-E253-255A. Total RNA was extracted at 7 hpi, and Northern blot was performed to detect IFN-β, ISG56 or RVFV S-RNA/N mRNA. None of those mutants attenuated NSs-mediated IFN-β mRNA synthesis suppression (**Fig. 8B**). These results suggest that attenuation of PKR degradation function might occur due to the accumulation of nonfunctional truncated NSs with some stability (**Fig. 3**) by the lack of C-terminus 17 amino acids residues.

Next, we tested whether the co-expression of NSΔ249–265 can inhibit MP-12 replication. A549 cells were infected with MP-12 using an moi of 0.01, and then were either mock-transfected or immediately transfected with *in vitro* synthesized RNA encoding CAT (RNA transfection control), NSΔ249–265 or NSs-E253-255A (a control with functional NSs). Culture supernatants were harvested at 72 hpi for viral titration using plaque assay. RVFV titer was significantly decreased by the CAT RNA transfection when compared to mock-transfection control. Transfection with RNA encoding NSΔ249–265 did not further decrease RVFV titer compared to CAT RNA transfected control, while RNA encoding NSs-E253-255A increased RVFV titer significantly. These results suggest that co-expression of NSΔ249–265 NSs does not significantly decrease viral replication, while that of NSs-E253-255A facilitates RVFV replication by inhibiting IFN-β and PKR in transfected cells. Overall, co-expression of truncated NSs inhibited neither NSs functions nor RVFV replication efficiently. Even though NSs encode self-association domain at the C-terminus domain, the expressed protein mislocalizes in cells, and does not maintain the stability of intact NSs, which minimizes the dominant-negative effect on MP-12 NSs.

## Discussion

Dominant-negative suppression of viral replication has been characterized in a number of different viral proteins [68], [69], [70], [71], [72], [73]. For RVFV, L proteins form oligomer, and exhibit dominant-negative function [74]. In this study, we used NSs protein as a target protein of dominant-negative suppression, because a lack of NSs dramatically attenuates RVFV [75], [76]. Since NSs can self-associate and form filamentous inclusion bodies in infected cells [65], [77], we hypothesized that co-expression of nonfunctional NSs with the C-terminal self-association domain in cells infected with RVFV allows incorporation of such nonfunctional NSs into the NSs filament, and attenuates a part of NSs functions. However, our results were not as expected; 1) most of truncated NSs localized at cytoplasm, 2) all of truncated NSs did not accumulate as much as parental NSs in cells, and 3) none of truncated NSs significantly interfered with MP-12 NSs functions. Our results suggest truncation of NSs causes mis-folding and/or mis-localization of protein, which might abolish the ability to interact with authentic MP-12 NSs through the C-terminus self-association domain. Unexpectedly, only the co-expression of NSs lacking C-terminus self-association domain (NSΔ249–265) slightly inhibited PKR degradation by MP-12 NSs. The NSΔ249–265 could accumulate both in cytoplasm and nucleus, which is consistent with previous study [65]. We speculate that NSΔ249–265 could compete with host factors required for PKR degradation with intact NSs. In the meantime, co-expression of NSΔ249–265 NSs did not result in a significant decrease of RVFV replication when compared to CAT RNA transfection control. Therefore, the co-expression of NSs fragments in infected cells might not be an effective strategy to inhibit RVFV replication in vivo.

Another novel finding is that all of truncation mutants; i.e., NSΔ6–30, NSΔ31–55, NSΔ56–80, NSΔ81–105, NSΔ106–130, NSΔ131–155, NSΔ156–180, NSΔ181–205, NSΔ206–230, NSΔ231–248 and NSΔ249–265, had lacked both PKR degradation and IFN-β suppression functions. This suggests that conformation structure might be important for those NSs functions rather than the presence of linear domain. Our results suggest that those truncated NSs decrease accumulation level, and change the localization pattern in cells. The stability and cellular localization of NSs, which are probably controlled by conformational domain, might be important for biological functions of NSs. Although the C-terminus 17 amino acids were determined as a self-association domain important for filament formation [65], our result suggests that filament formation does not occur by NSs encoding an in-frame truncation with 25 amino acids. It is possible that the C-terminus 17 amino acids are the prerequisite of NSs self-association, and other structural domains play a role in the filament formation through the C-terminus domain.

Our result showed that NSΔ206–230 dominantly localized at cytoplasm. Previous study showed that rec-ZHΔ210–230 (recombinant ZH548 encoding an in-frame truncation in NSs at 210–230) could induce IFN-β mRNA due to a lack of SAP30-binding domain in infected cells [26]. It was also shown that NSs binding to SAP30 is required for NSs filaments to target pericentromeric DNA and induce nuclear anomalies [78]. They described that rec-ZHΔ210-230 expresses a stable NSs protein located in the nucleus in the discussion [78]. Thus, it might be possible that NSs encoding 20 aa. does not change the nuclear localization. On the other hand, NSΔ6–30, NSΔ31–55, NSΔ56–80 and NSΔ249–265, all of which encode SAP30-binding domain at aa.210–130, could be accumulated in nucleus, whereas they did not inhibit IFN-β gene, suggesting that NSs has another structural requirement to inhibit IFN-β gene activation in addition to SAP30 binding. The requirement of NSs localization and IFN-β gene suppression should be further studied to understand the detailed mechanism of IFN-β gene regulation by RVFV NSs.

We also characterized the role of C-terminus acidic residues in NSs functions. We found that both NSs-E253-255A/D257-259A and NSsD257-259A were not abundantly accumulated in infected cells. On the other hand, the NSs-E253-255A accumulated efficiently in cells, and showed a similar phenotype with authentic NSs. Thus, the aspartic acids at 257–259 but not glutamic acid at 253–255 must be important for the stability of NSs. Since the accumulation of NSs-E253-255A/D257-259A and NSsD257-259A were very low, we could not determine whether those mutants are lacking the functions to degrade PKR or inhibit IFN-β mRNA synthesis.

We noted that the co-infection of recombinant MP-12 encoding truncated NSs with MP-12, except for NSΔ6–30 and NSΔ56–80, had resulted in dominant accumulation of truncated NSs. This effect may possibly occur at the transcription or translation level rather than post-translation level, since MP-12 NSs can also accumulate with truncated NSs when RNA transfection was used for truncated NSs expression. If a selective viral transcription of truncated NSs mRNA, or a selective translation of truncated NSs proteins could occur in co-infected cells, then a virus exhibiting these traits may be useful for post-exposure vaccination. However, new studies will be required to detail this mechanism.

In summary, short in-frame truncations of RVFV NSs affect the expression level and cellular localization, which lessen or abolish biological functions of NSs most probably due to the lack of functional conformation domains. Thus, co-expression of truncated nonfunctional NSs in RVFV-infected cells does not attenuate NSs functions of RVFV.

## Materials and Methods

### Media, Cells and Viruses

VeroE6 cells (ATCC CRL-1586), 293 cells (ATCC CRL-1573) and A549 cells (ATCC CCL-185) were maintained in Dulbecco's modified minimum essential medium (DMEM) containing 10% fetal calf serum (FCS). BHK/T7–9 cells that stably express T7 RNA polymerase [79] were maintained in MEM-alpha containing 10% FCS. Penicilin (100 U/ml) and streptomycin (100 g/ml) were added to the culture media.

### Plasmids

The plasmid encoding anti-viral-sense of MP-12 S-segment at the downstream of the T7 promoter, pProT7-S(+), was described previously [18]. Serial deletion of 75 bp (25 aa.) was introduced into the NSs open reading frame (ORF) of pProT7-S(+) by site-directed mutagenesis with Pfu Turbo DNA polymerase (Stratagene), designated as pProT7-S(+)-NSΔ6–30, NSΔ31–55, NSΔ56–80, NSΔ81–105, NSΔ106–130, NSΔ131–155, NSΔ156–180, NSΔ181–205, NSΔ206–230 or NSΔ231–248, respectively. For C-terminus mutant, the PCR fragment encoding NSs ORF with C-terminus 51 bp (17 aa.) deletion was amplified, and cloned between *Hpa*I and *Spe*I of pProT7-S(+) [18], designated as NSΔ249–265. The alanine substitutions of NSs-E253-255A/D257-259A, NSs-E253-255A or NSsD257-259A were made onto pProT7-S(+) plasmid by site-directed mutagenesis with Pfu Ultra (Agilent Technologies), and designated as pProT7- NSs-E253-255A/D257-259A, NSs-E253-255A or NSsD257-259A, respectively. NSs ORF of those NSs mutants were amplified by PCR with Phusion High Fidelity DNA polymerase (New England Biolab), and cloned into pcDNA3.1mycHisA (Invitrogen) between KpnI and XhoI, and designated as pcDNA3.1mycHisA- NSΔ6-30, NSΔ31–55, NSΔ56–80, NSΔ81–105, NSΔ106–130, NSΔ131–155, NSΔ156–180, NSΔ181–205, NSΔ206–230, NSΔ231–248, NSΔ249–265, NSs-E253-255A/D257-259A, NSs-E253-255A or NSsD257-259A.

### Recovery of recombinant MP-12

The recombinant MP-12 encoding NSs truncation or mutation were recovered by using a plasmid combination of pProT7-M(+), pProT7-L(+), pT7-IRES-vN, and pT7-IRES-vL and either of pProT7-S(+)-NSΔ6–30, NSΔ31–55, NSΔ56–80, NSΔ81–105, NSΔ106–130, NSΔ131–155, NSΔ156–180, NSΔ181–205, NSΔ206–230, NSΔ231–248, NSΔ249–265, NSs-E253-255A/D257-259A, NSs-E253-255A or NSsD257-259A. BHK/T7-9 cells were transfected with those plasmids as described previously [18].

### Northern blot analysis

Total RNA was extracted from infected or mock-infected cells using TRIzol reagent. Denatured RNA was separated on 1% denaturing agarose-formaldehyde gels and transferred onto a nylon membrane (Roche Applied Science). Northern blot analysis was performed as described previously with strand-specific RNA probes to detect RVFV anti-sense S-segment/N mRNA, human IFN-β mRNA, or human ISG56 mRNA [66], [67].

### Western blot analysis

Western blot analysis was performed as described previously [17]. The membranes were incubated with anti-PKR monoclonal antibody (BD biosciences), anti-RVFV mouse polyclonal antibody (a kind gift from Dr. R.B.Tesh, UTMB), anti-NSs rabbit polyclonal antibody [66], anti-Flag-tag M2 monoclonal antibody (Sigma), or anti-actin goat polyclonal antibody (I-19; Santa Cruz Biotech.) overnight at 4°C and with secondary antibodies for 1 hr at room temperature.

### Analysis of virus replication

A549 cells were infected with rMP12-NSs-Flag at an moi of 0.01, and mock-transfected or immediately transfected with in vitro synthesized RNA encoding CAT, NSΔ249-265 or NSs-E253-255A. At 72 hpi, culture supernatants were collected, and plaque assay was performed as described previously [18], [80].

### In vitro RNA synthesis

The pcDNA3.1mycHisA plasmids encoding CAT [17], NSΔ6–30, NSΔ31–55, NSΔ56–80, NSΔ81–105, NSΔ106–130, NSΔ131–155, NSΔ156–180, NSΔ181–205, NSΔ206–230, NSΔ231–248, NSΔ249–265, NSs-E253-255A/D257-259A, NSs-E253-255A or NSsD257-259A were linearized, and in vitro transcribed by using mMESSAGE mMACHINE T7 Ultra kit (Ambion) according to manufacturer's instruction. The linearized CAT DNA contained myc-His tag at the 3′end.

### Transfection

Transfection of in vitro synthesized RNA was performed by using TransIT-mRNA Transfection Kit (Mirus) according to manufacturer's instruction.

### Separation of nuclear and cytoplasmic fractions

VeroE6 cells were mock-infected or infected with MP-12 or recombinant MP-12 encoding partially truncated NSs at an moi of 4 in 6-well plate. At 16 hpi, cells were collected, and washed once in PBS. Then, cytoplasmic fraction was lyzed with PBS containing 1% TritonX-100 on ice for 5 min. After centrifugation at 10,000 xg at 4^°^C for 5 min, nuclear fraction was washed once with cold PBS, and resuspended in PBS containing 1% TritonX-100. Both cytoplasmic and nuclear fractions were mixed with 2× SDS sample buffer, and subjected to SDS-PAGE and Western blot analysis.

### Co-affinity precipitation

SF (Strep-Flag)-tagged protein was precipitated with Strep-Tactin magnetic beads (Qiagen). 293 cells were first infected with rMP-12 tagged with SF-tag (moi 3) and were then transfected with *in vitro* synthesized capped RNA (encoding NSΔ6–30, NSΔ31–55, NSΔ56–80, NSΔ81–105, NSΔ106–130, NSΔ131–155, NSΔ156–180, NSΔ181–205, NSΔ206–230, NSΔ231–248, or NSΔ249–265). After incubation for 6 hours, newly synthesized proteins were then labeled with [^35^S] methionine/cysteine (MP Biomedicals). Using cell lysates, SF-tagged proteins were precipitated with Strep-Tactin beads according to manufacturer's instructions. Then, co-precipitated proteins were analyzed by separating on 10% SDS-PAGE gel and followed by autoradiography.

### Statistical analysis

Unpaired Student's t-test was performed by using the Graphpad Prism 5.03 program (Graphpad Software Inc.) for the comparison of two groups,

### Ethics statement

All the recombinant DNA and RVFV were created upon the approval of the Notification of Use by the Institutional Biosafety Committee at UTMB.

## Supporting Information

Figure S1 **Plaque phenotypes of MP-12 encoding NSs mutants.** VeroE6 cells were infected with indicated virus as 10-fold dilution, and overlaid with 0.6% noble agar containing 5% FBS and 5% Tryptose phosphate broth in MEM as described previously [80]. Second overlay of agar containing 0.011% of neutral red was performed at 3 dpi. Plaques at 4 dpi are shown.
(TIF)

Figure S2 **Titer of MP-12 NSs mutants in MRC-5 cells.** Human lung diploid MRC-5 cells were infected with MP-12, rMP12-C13type (C13type) or NSs mutants encoding indicated truncations at an moi of 0.01. At 72 hpi, culture supernatants were collected, and virus titers were measured by plaque assay using VeroE6 cells. Means and standard deviations of 3 independent experiments are shown. **p<0.01, Student's unpaired t-test compared to MP-12.
(TIF)

Figure S3 **Co-infection of MP-12 and MP-12 encoding truncated NSs.** VeroE6 cells were mock-infected or infected with a mixture of MP-12 (an moi of 3) and either of rMP12-C13type (C13type) or indicated NSs truncation mutants (an moi of 3). Cells were collected at 16 hpi, and PKR (anti-PKR antibody), NSs and N (anti-RVFV antibody) and β-actin (anti-actin antibody) were detected by Western blot.
(TIF)
