# Supplementary figures and images for: Functional Analysis of Rift Valley Fever Virus NSs Encoding a Partial Truncation
Source: PLoS One. 2012 Sep 19;7(9):e45730. doi: 10.1371/journal.pone.0045730 (PMC3446906; doi:10.1371/journal.pone.0045730)

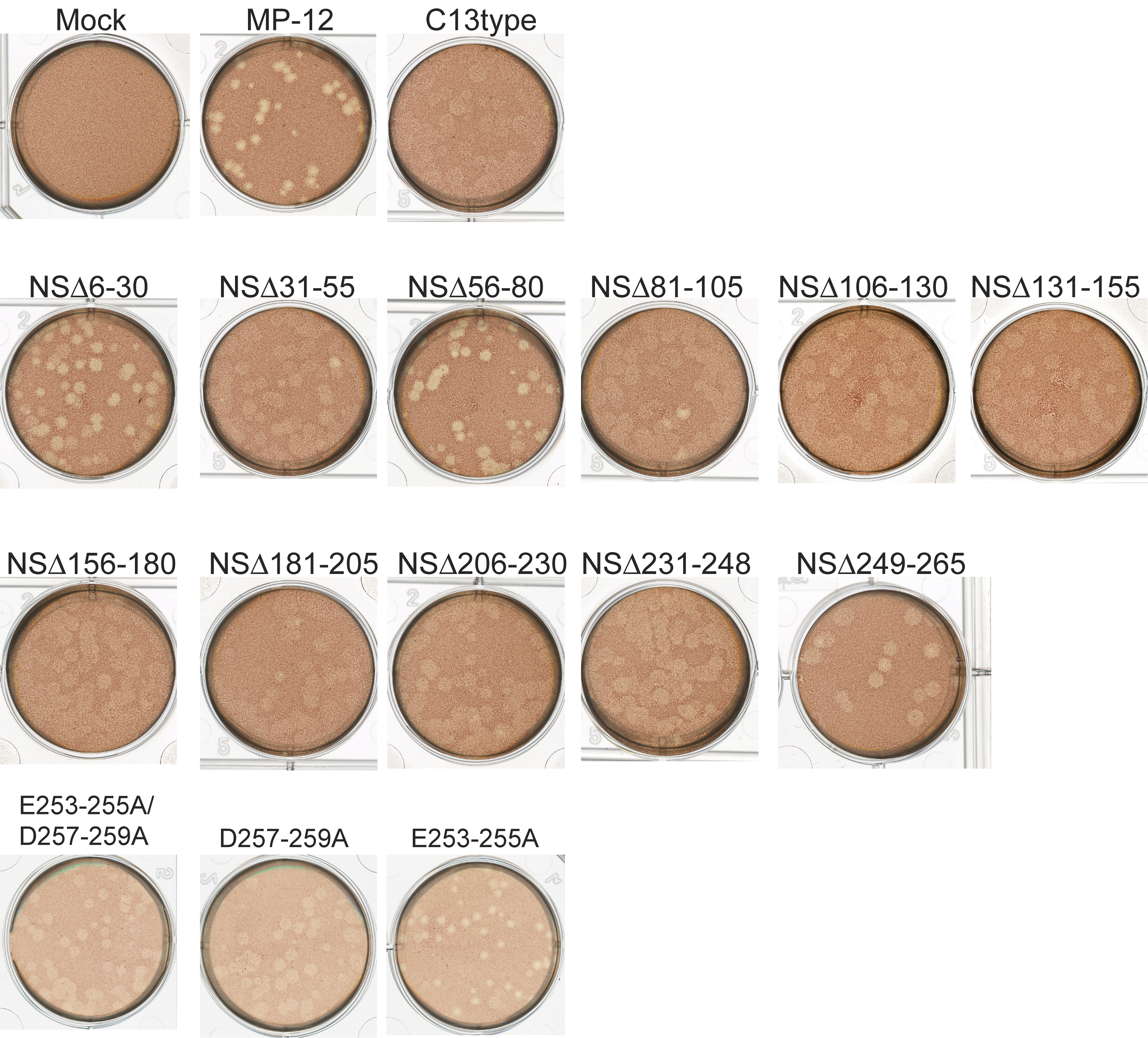

Supplement: Figure S1 — Plaque phenotypes of MP-12 encoding NSs mutants. VeroE6 cells were infected with indicated virus as 10-fold dilution, and overlaid with 0.6% noble agar containing 5% FBS and 5% Tryptose phosphate broth in MEM as described previously [80]. Second overlay of agar containing 0.011% of neutral red was performed at 3 dpi. Plaques at 4 dpi are shown. (TIF) [file pone.0045730.s001.tif]

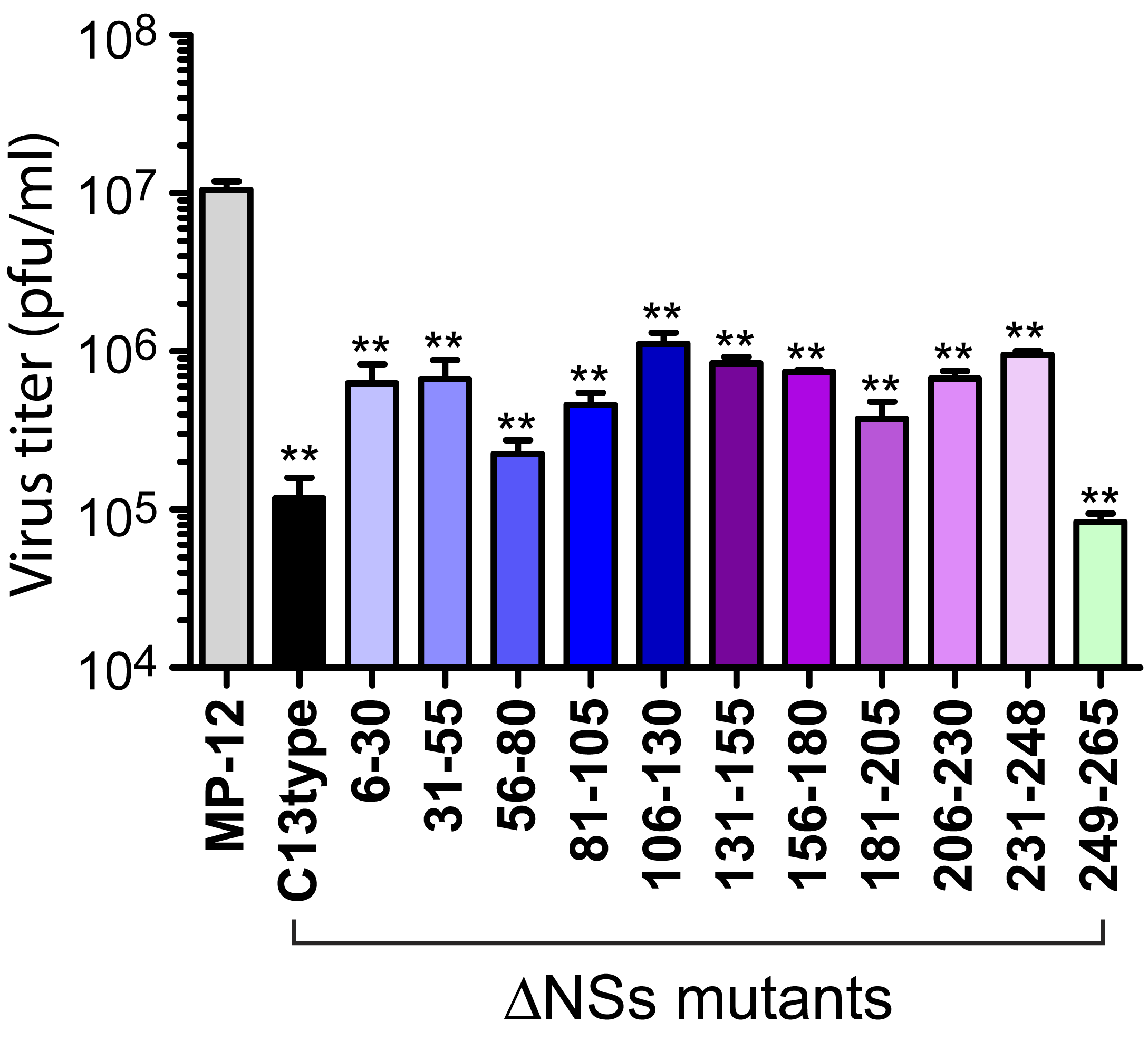

Supplement: Figure S2 — Titer of MP-12 NSs mutants in MRC-5 cells. Human lung diploid MRC-5 cells were infected with MP-12, rMP12-C13type (C13type) or NSs mutants encoding indicated truncations at an moi of 0.01. At 72 hpi, culture supernatants were collected, and virus titers were measured by plaque assay using VeroE6 cells. Means and standard deviations of 3 independent experiments are shown. **p<0.01, Student's unpaired t-test compared to MP-12. (TIF) [file pone.0045730.s002.tif]

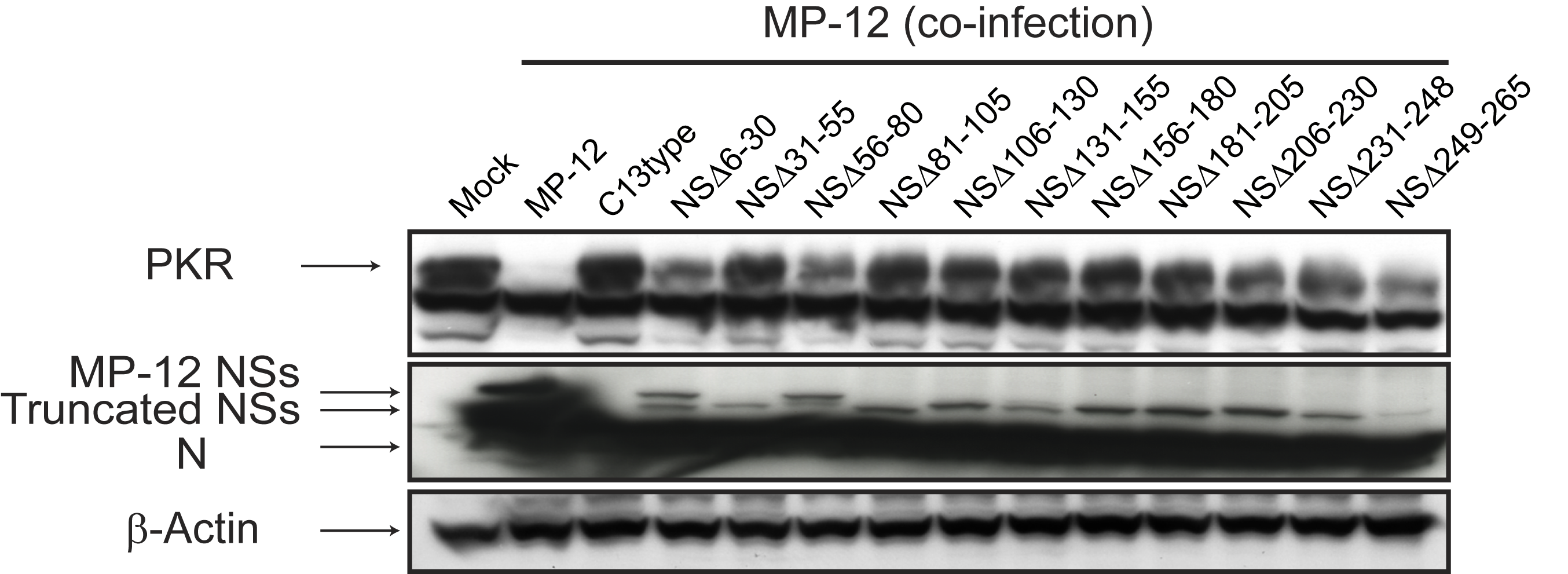

Supplement: Figure S3 — Co-infection of MP-12 and MP-12 encoding truncated NSs. VeroE6 cells were mock-infected or infected with a mixture of MP-12 (an moi of 3) and either of rMP12-C13type (C13type) or indicated NSs truncation mutants (an moi of 3). Cells were collected at 16 hpi, and PKR (anti-PKR antibody), NSs and N (anti-RVFV antibody) and β-actin (anti-actin antibody) were detected by Western blot. (TIF) [file pone.0045730.s003.tif]
